# Effect of a single rectal fecal microbiota transplantation on clinical severity and fecal microbial communities in dogs with chronic inflammatory enteropathy

**DOI:** 10.1111/jvim.17264

**Published:** 2025-01-08

**Authors:** Jorge Pérez‐Accino, Mazdak Salavati, Laura Glendinning, Silke Salavati Schmitz

**Affiliations:** ^1^ College of Medicine and Veterinary Medicine, The Royal (Dick) School of Veterinary Studies, Hospital for Small Animals, Easter Bush Campus University of Edinburgh Midlothian UK; ^2^ South and West Faculty, Dairy Research Innovation Centre Scotland's Rural College Dumfries UK; ^3^ College of Medicine and Veterinary Medicine, The Roslin Institute, Genetics and Genomics Department, Easter Bush Campus University of Edinburgh Midlothian UK; ^4^ Present address: Hospital Canis Girona Spain

**Keywords:** bacteria, diarrhea, inflammatory bowel disease, microbiome, transfaunation

## Abstract

**Background:**

Fecal microbiota transplantation (FMT) has been advocated as a treatment for chronic enteropathy (CE) in dogs. However, so far only short‐term clinical effects have been reported whereas the effect on the microbiota remains unexplored.

**Hypothesis/Objectives:**

Assess if a single FMT enema can lead to clinical improvement in dogs with CE when accompanied by presumed favorable microbiota changes. The effect of glycerol as a cryopreservative when storing FMT preparations also was assessed.

**Animals:**

Seven dogs with CE that received FMTs from 2 healthy donor dogs.

**Materials and Methods:**

Six dogs received a single FMT, 1 dog received 3 consecutive FMTs. Canine chronic enteropathy clinical activity index (CCECAI) and fecal samples were obtained before (Day 0), and 7, 30 and 90 days after FMT. Samples were stored with and without 10% glycerol. Sequencing of microbiota (16S rRNA, Illumina) was performed and compared by accepted analysis pipelines.

**Results:**

Median CCECAI before FMT was 8 (range, 5‐14), decreased to a median of 3 (range, 1‐12) within 1 week and a median of 1 (range, 0‐12) by Day 30 (*P* < .01), with an average duration of response of approximately 10 weeks. Significant variation in the donors' microbiota composition was observed across different donations. Recipient microbiota composition or diversity did not change over time. Glycerol addition was associated with a difference in microbiota composition (*P* ≤ .001).

**Conclusions and Clinical Importance:**

A single FMT can be considered an appropriate treatment in dogs with CE, but consistent microbiota changes were not observed.

**Abbreviations**
bp: base pair(s)
BW: body weight
CCECAI: canine chronic enteropathy clinical activity index
CD: Crohn's disease
CE: chronic enteropathy
DI: dysbiosis index
FMT: fecal microbiota transplantation
GI: gastrointestinal
NMDS: nonmetric multidimensional scaling
NRE: nonresponsive chronic enteropathy
OTU: observed taxonomical units
PERMANOVA: permutational multivariate analysis of variance
PFS: Purina fecal score
rRNA: ribosomal ribonucleic acid
UC: ulcerative colitis

## 1 INTRODUCTION

Chronic enteropathy (CE) is a prevalent chronic gastrointestinal (GI) inflammatory condition in dogs with no single cure and its similarity to inflammatory bowel disease in people is still debated.^1^ Chronic enteropathy encompasses a group of diseases that have variable clinical presentations and is considered a descriptive diagnosis requiring exclusion of other GI and extra‐GI conditions.^1^ Subclassification traditionally has been done retrospectively based on response to step‐wise empirical treatments such as elimination dietary trials, antibiotics, and immunosuppressants.^1^ However, nomenclature for this condition and its subtypes frequently has been challenged in recent years,^1^, ^2^ as has the justification for some empirical treatments. Great strides have been made in understanding the multifactorial pathogenesis of CE, particularly regarding the role of the intestinal microbiota and its interaction with the host immune system, and novel treatment options have been sought.^2^ In addition, 15% to 43% of dogs with CE have nonresponsive enteropathy (NRE^1^), with poor long‐term prognosis and high risk of euthanasia.^3^, ^4^ Antibiotics are no longer considered appropriate standard of care for CE because of their detrimental effects on the microbiota,^5^, ^6^ and new treatments should be tailored toward microbiota restoration and health, rather than solely assessing clinical responses.^2^, ^6^

A recent meta‐analysis demonstrated that the majority of dogs with CE have abnormal intestinal microbiota (ie, dysbiosis)^7^ characterized by loss of microbial diversity and decreased abundance of *Faecalibacterium*, *Fusobacterium*, *Blautia*, *Turicibacter*, and *Clostridium* (now *Peptacetobacter*) *hiranonis* as well as increased abundance of *Escherichia (E.) coli* and streptococci.^8^, ^9^ Despite these findings, microbiota‐targeting interventions for CE still are considered novel or alternative and have not yet achieved the status of established treatments. This situation is likely because of the paucity of evidence for their use in alleviating clinical signs or improving intestinal dysbiosis.

Fecal microbiota transplantation (FMT) is considered a relatively novel adjunctive treatment option for CE, which in principle could directly address dysbiosis, and is more likely to induce clinically relevant microbiota changes compared with administration of pro‐ or prebiotics, which have not had a substantial effect on the microbiota of dogs.^10^ Fecal microbiota transplantation has been used successfully in people with ulcerative colitis (UC) and Crohn's disease (CD) with a recent Cochrane meta‐analysis concluding that FMT may increase the proportion of UC patients achieving clinical remission.^11^

In dogs, FMT has been clinically beneficial in acute and infectious GI diseases, such as parvovirosis, where FMT significantly decreased time to recovery and duration of hospitalization.^12^ Fecal microbiota transplantation was superior to metronidazole in correcting intestinal dysbiosis within 28 days of an episode of acute diarrhea in dogs, with a comparable clinical response.^6^ Until recently, however, little has been published on the effectiveness of FMT in dogs with CE, both from a clinical as well as microbiota perspective. In 1 prospective study, a clinical response to FMT was noted in 20/27 dogs with CE, as documented by a significant decrease in the canine chronic enteropathy clinical activity index (CCECAI^4^) 15 days after a month‐long treatment with daily PO freeze‐dried FMT capsules.^13^ Clinical outcome beyond 15 days was not reported. The largest study on FMT in dogs with CE reported a clinical response in 31/41 dogs with NRE.^14^ In this study, information on microbiota before rectal FMT was available for 16 dogs in the form of the commercially available dysbiosis index (DI) test.^15^ This qPCR based assay (quantification of *Blautia* sp., *Bacteroides* sp., *Bifidobacerium* sp., *Clostridium hiranonis, E. coli, Faecalibacterium prausnitzii, Fusobacterium* sp., *Streptococcus* sp., and *Turicibacter* sp.) has been shown to significantly differ between healthy dogs and dogs with CE,^16^ and was predictive of the clinical response after FMT.^14^ However, no follow‐up DI or other microbiota assessment of the recipients was available after FMT in that study.

Our study was conducted to examine the hypothesis that a single FMT enema can lead to clinical improvement in dogs with CE, and that it will be driven by microbiota changes that will allow FMT recipients to experience short‐lived changes in their microbiome profile that resemble those of healthy FMT donors.

As secondary objectives, microbiota differences from individual donors across repeated donations, as well as the effect of processing and storage of FMT (eg, with and without glycerol as a cryopreservative) were assessed.

## 2 MATERIALS AND METHODS

### 2.1 Animals and clinical scores

The FMT recipients were dogs presented to the Internal Medicine Referral service of the Hospital for Small Animals at the University of Edinburgh for chronic (≥3 weeks) GI signs and with a final diagnosis of CE. This diagnosis included ruling out other causes of GI signs by performing a CBC, serum biochemistry, fecal flotation or sedimentation or both and *Giardia* sp. antigen testing, basal plasma cortisol concentration or ACTH‐stimulation test, abdominal ultrasonography and GI endoscopy with mucosal biopsies. Dogs must have undergone at least 1 reasonable attempt to treat CE with an elimination diet trial of 2 to 4 weeks' duration, before pet caretakers were offered participation in the study. Written consent was obtained from all dog owners or authorized caretakers, and the study was approved by the university's veterinary ethical review committee (VERC#117.16). Dogs were not allowed to receive antibiotics or probiotics in the 2 weeks before FMT (wherever possible all other medications also were discontinued) and did not receive any other treatments during the observation period after receiving FMT. In all dogs, CCECAI^4^ scores (based on a standardized owner questionnaire) were determined before FMT and 7 and 30 days thereafter. Samples from the same time points (and additional samples 90 days after FMT when available) were brought to the hospital on the same day of passage, cooled on transport, and aliquots were stored with and without 10% glycerol at −20°C until processing for microbiota analysis.

### 2.2 Fecal microbiota transplantation donors, processing, storing, and administration

The FMT donors were 2 selected healthy adult dogs owned by members of staff with ideal body condition. They had no history of any disease including GI disease, were not on any medications or supplements, were not allowed to have received antimicrobials for any reason in the 12 months before donating and had to be fed a commercial complete and balanced dog food. Raw food feeding or home‐cooked food was not permitted. They were regularly treated for endo‐ and ecto‐parasites and before every donation tested for pathogens by fecal flotation or sedimentation or both and *Giardia* sp. antigen testing as well as selective fecal culture for Salmonella, Yersinia, and Campylobacter. Their feces were scored by the Purina fecal score (PFS; ideal score: 2‐4) before every donation and rejected if considered inappropriate consistency or if containing visible foreign materials.

For each donation, a fresh fecal sample was received and processed within 4 hours of passage (if necessary, it was kept cool but not frozen for transport to the hospital). Upon receipt, the sample was weighed and mixed with sterile saline at a ratio of 1 : 5 by a household blender. If necessary, any coarse material was filtered out using a colander or muslin cheesecloth. The resulting FMT slurry was transferred into 50 mL syringes and capped. These were stored at 4°C until use (<6 hours from the start of processing).

Aliquots of all donor samples at each donation as well as the FMT slurry after each processing were frozen at −20°C with and without 10% glycerol for later microbiota analysis. The donor utilized for each recipient was determined by donor availability.

The FMT was administered to each recipient as a rectal enema by a soft polyvinyl catheter, at a dosage of 30 mL/kg body weight (BW) of the recipient. A warm water enema was given beforehand to all dogs to remove as much residual recipient feces as possible if necessary. The recipients were conscious and standing; mild sedation using 0.3 mg/kg BW of butorphanol IV was allowed if needed. The FMT was retained in place for 15 minutes before the dog was allowed to move freely. The entire procedure was done at an outpatient appointment and the FMT recipient was discharged afterwards.

### 2.3 Statistical analysis of clinical data

The CCECAI scores were compared by GraphPad Prism 9.5.1 for Windows, GraphPad Software, San Diego, California (www.graphpad.com). Data were tested for normality by Shapiro‐Wilk tests and compared by Friedman test with multiple comparisons. Because of the small sample size, the proportion of dogs is expressed by a 95% binomial confidence interval to provide an estimate of the true population proportion.

### 2.4 Fecal DNA extraction, 16S rRNA gene amplification, and sequencing

The DNA extraction was performed as described previously^17^ with the DNeasy PowerLyzer PowerSoil Kit (Qiagen).

A 390‐base segment of the V4 hypervariable region of the 16S rRNA gene was amplified by the universal primers 515f and 806r following a previously described method.^18^ The reaction mix contained 12.5 μL of Q5 High Fidelity DNA Polymerase (New England Biolabs), 1.25 μL each of custom 10 nM forward and reverse primers with Illumina adaptor sequences, spacers, and barcodes, 9 μL of nuclease‐free water (Qiagen), and 1 μL of DNA template. Cycling conditions were as follows: 95°C for 2 minutes; followed by 30 cycles of 95°C for 20 seconds, 55°C for 15 seconds and 72°C for 5 minutes; followed by a final extension step at 72°C for 10 minutes. No‐template controls were used alongside every sample. Amplicons were purified with the AMPure XP System (Beckman Coulter), according to the manufacturer's instructions, except that a 1 : 1 ratio of AMPure beads to sample was used. The amplicon concentrations were determined with the Qubit 3.0 fluorometer (Life Technologies) with the Qubit dsDNA HS Assay Kit (Life Technologies), according to the manufacturer's instructions. Samples then were pooled into an equimolar library, and sequenced on the Illumina MiSeq platform by Edinburgh Genomics (Edinburgh, UK).

### 2.5 Quality control, alignment and taxonomic assignment of 16S rRNA reads, and sequence analysis

Mothur^19^ was used for quality control of sequences, alignment, taxonomic assignment and observed taxonomical units (OTU) clustering, following a modified version of the MiSeq pipeline^18^ supplied on the mothur website. Briefly, sequences were removed if they were >254 base pair (bp) in length, contained ambiguous bases, had homopolymers >9 bp in length, did not align to the V4 region of the 16S rRNA gene or did not originate from bacteria. Chimeras were identified by VSEARCH (v.2.15.2)^20^ and were removed. The SILVA database (release 138.1)^21^ was used for sequence alignment and taxonomic assignment. The OTUs were clustered by the mothur commands cluster.split (taxlevel = 4, cutoff = 0.03), and make.shared (label = 0.03).

Statistical and graphical analyses of microbiota data were conducted using R (v.4.2.2).^22^ The R packages used during analyses included ANCOMBC^23^, ^24^ (v.2.0.3), cowplot^25^ (v.1.1.1), dplyr^26^ (v.1.1.1), ggplot2^27^ (v.3.4.2), phyloseq^28^ (v.1.42.0), stringr^29^ (v.1.5.0), tidyr^30^ (v.1.3.0), and vegan^31^ (v.2.6.4). Nonmetric multidimensional scaling (NMDS) graphs were constructed using values produced by metaMDS from the vegan package, using Bray‐Curtis dissimilarity values. Permutational multivariate analysis of variance (PERMANOVA) was conducted using Bray‐Curtis dissimilarity values, and the adonis2 command from the vegan package. Alpha diversity richness and diversity indices were calculated by the vegan commands estimateR (Chao1 richness estimator) and diversity (Inverse Simpson's diversity index), and tested for significance by the Kruskal‐Wallis test. Analysis of compositions of microbiomes (ANCOMBC),^32^ with Benjamini‐Hochberg corrections, was used to identify specific taxa that were differentially abundant between groups.

## 3 RESULTS

### 3.1 Animals and clinical scores

**FIGURE 1:**
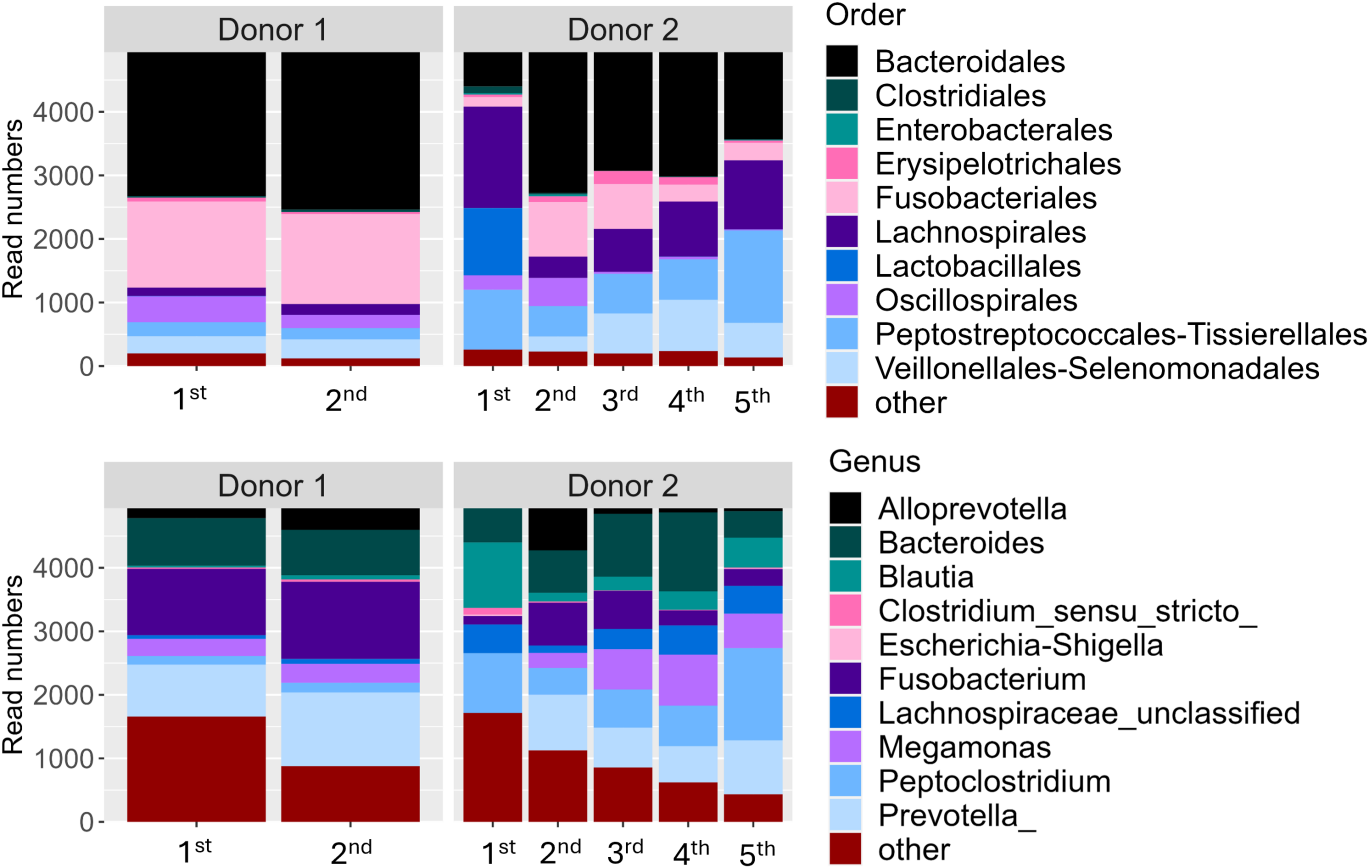
Microbiota composition of the 2 FMT donor dogs for this study. The upper 2 panels show predominant bacterial orders, and the lower 2 panels predominant bacterial genera.

Seven dogs with CE completed the study between May 2017 and January 2019 as FMT recipients. Their signalments and main clinical characteristics are found in Table S1. Median CCECAI before FMT was 8 (range, 5‐14). Donor 1 donated twice and the resulting FMT was given to 3 dogs. Donor 2 donated 4 times and the resulting FMTs were given to the remaining 4 dogs. The donors' microbiota composition and variation across different donation days are presented in Figure 1. Because of individual differences in microbiota composition among donors and for each donor, further microbiota comparisons of recipients were done with their matching donation only.

**FIGURE 2:**
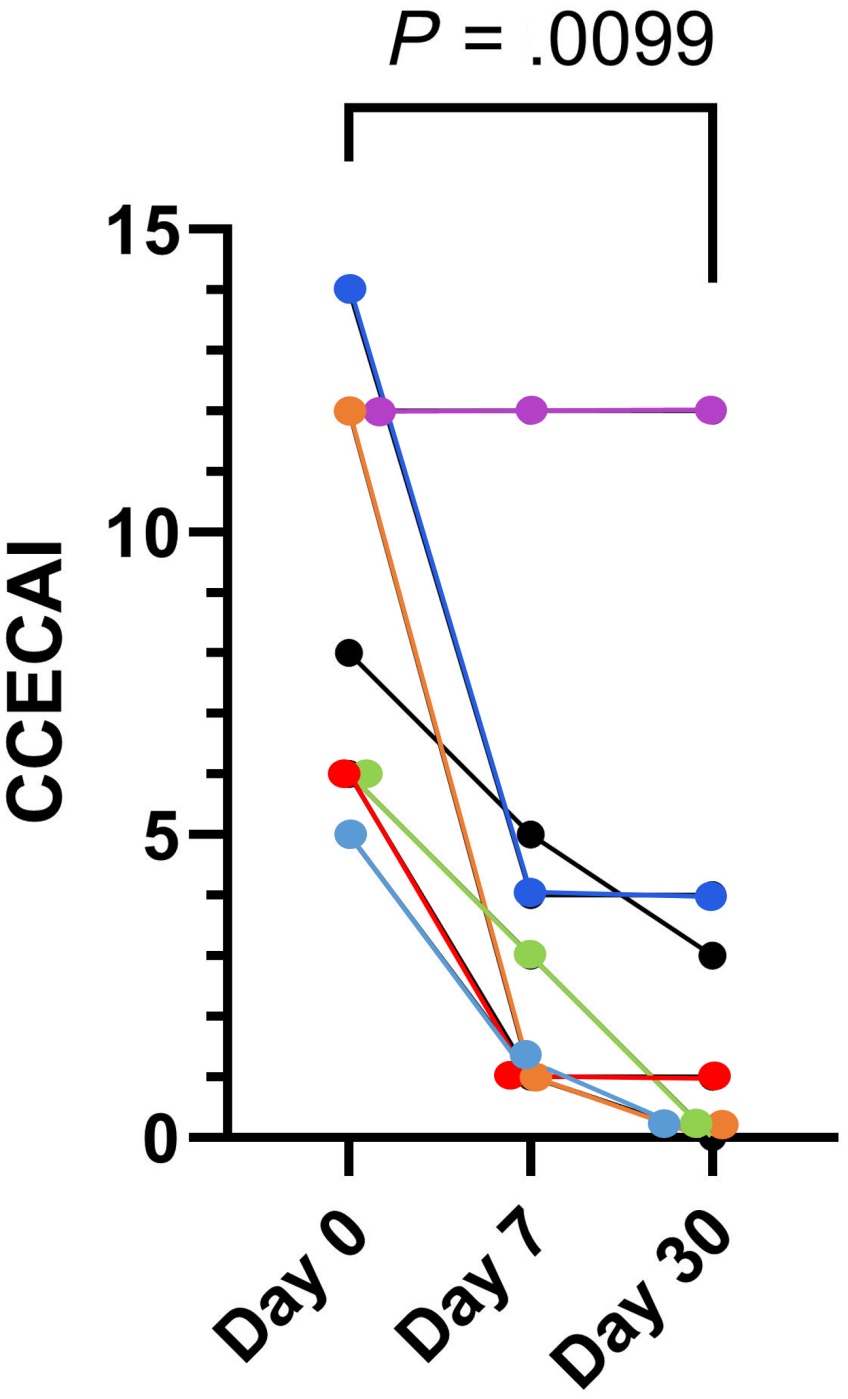
Canine chronic enteropathy clinical activity index (CCECAI) in 7 dogs before, 7 and 30 days after receiving a single rectal FMT.

In 6/7 (85.7%, 95% confidence interval [CI]: 42.1%‐99.6%) recipient dogs CCECAI decreased to a median of 3 (range, 1‐5) within 1 week of a single FMT (*P* = .18). It then further decreased to a median of 1 (range, 0‐4) in 4/7 (57.1%; 95% CI: 18.4%‐90.1%) dogs, which was significant (*P* < .01) by Day 30 (Figure 2). In Dog 4, CCECAI remained static at a score of 12 (severe). Overall, clinical improvement for the remaining 6 dogs lasted a median of 73 days (range, 30‐1168 days). Dog 7 received 3 FMT procedures when clinical relapses occurred, and responses lasted 85, 28, and 113 days, respectively. Follow‐up was available for a median of 420 days (range, 48‐3024 days). After FMT, only 1/7 dogs (14.3%; 95% CI: 0.4%‐57.9%; Dog 6) remained clinically well longer‐term on diet management alone. Dog 1 required intermittent symptomatic treatment for abdominal pain (butylscopolamine and paracetamol) but was euthanized because of metastatic cutaneous hemangiosarcoma 3 months later. One dog remained well on diet with intermittent courses of tylosine, 1 each responded well subsequently to prednisolone or tylosine alone, respectively, and 2 continued to be nonresponsive to any administered treatment. These 2 dogs had the highest clinical activity scores of the cohort (CCECAI of 12 at Day 0). Of those, Dog 2 was managed with intermittent courses of metronidazole, prednisolone, cyclosporine, and different diets, and is still alive at the time of writing (3024 days after FMT). The other dog (Dog 4) had a marginal and short‐lived response to prednisolone, worsened on cyclosporine and was euthanized 48 days after FMT, having been refractory to treatment. All of these additional treatments were begun after the last sampling for the study, and the study was considered terminated if other treatments were deemed necessary by the attending clinicians.

### 3.2 Effect of glycerol on fecal material storage

**FIGURE 3:**
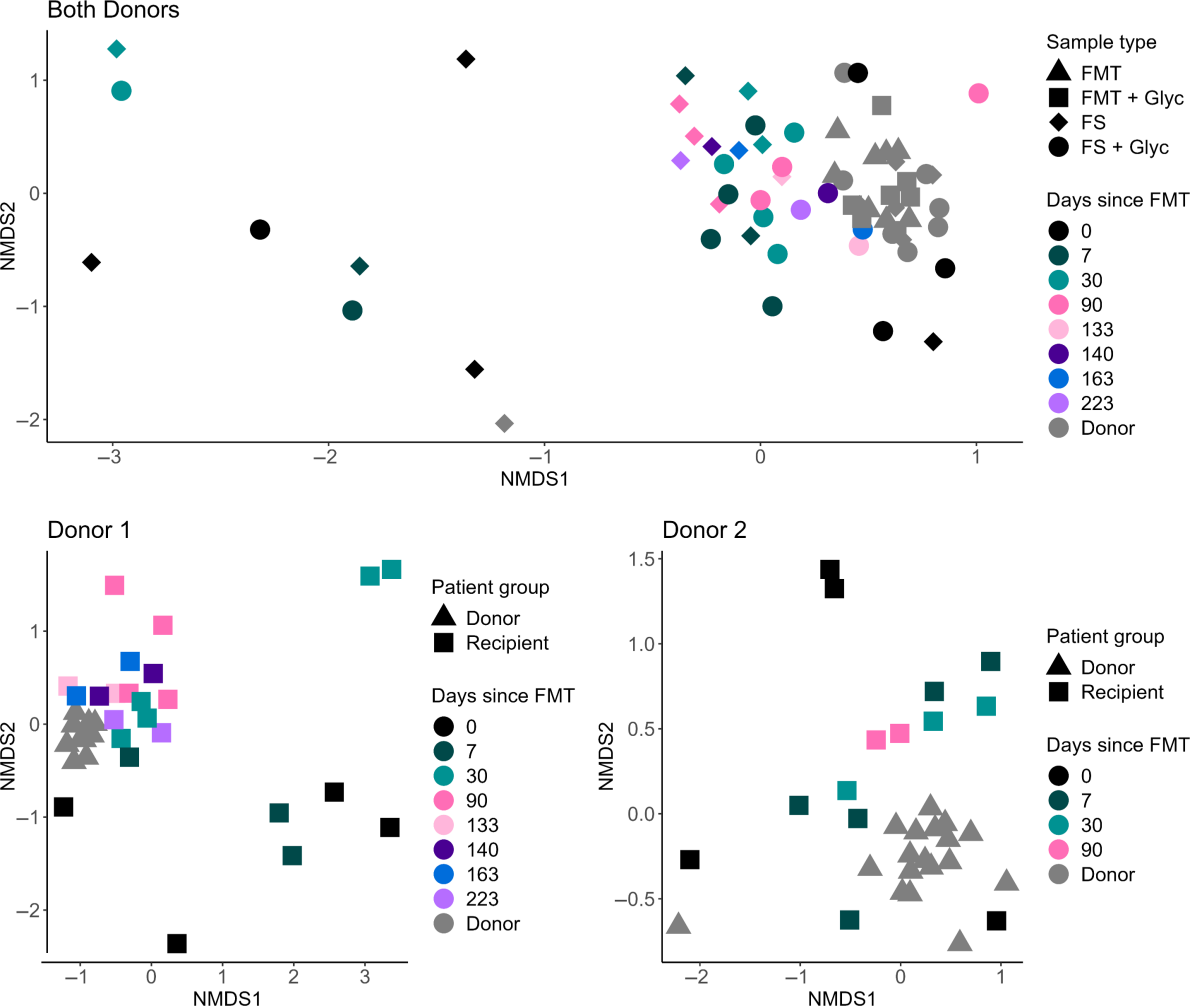
NMDS clustering of samples using Bray‐Curtis dissimilarity values (stress = 0.18). Different colors indicate days since FMT administration, donor samples are gray. Upper panel: All samples from both donors and all recipients, by sample type (indicated by different shaped symbols): FMT, fecal microbiota transplant slurry; FS, fecal sample before processing; Glyc, with the addition of 10% glycerol. Lower panels: Samples from all FMT recipients from donor 1 (left) and donor 2 (right). Donors are represented by (gray) triangles and recipients by squares.

**FIGURE 4:**
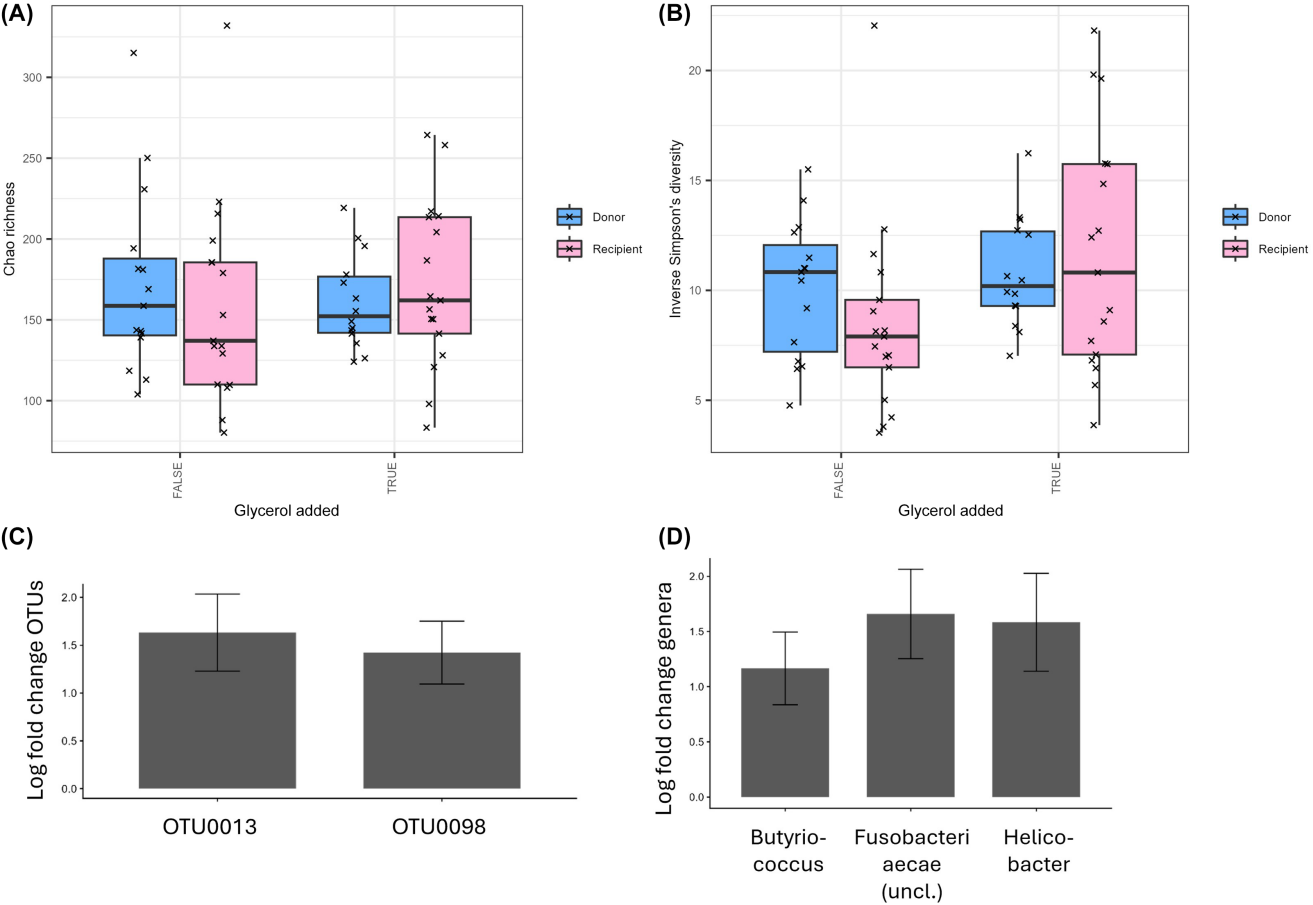
Upper panels: Boxplots showing richness (A: Chao1 Index) and diversity (B: Inverse Simpson's index) of fecal samples from FMT donor and recipient dogs with or without the addition of glycerol. Lower panels: Log fold changes in OTUs (C) and genera (D) that were defined as significantly differently abundant by ANCOMBC2 (*q* < 0.05) between samples with and without glycerol. Error bars represent standard errors.

Beta diversity (quantification of the similarity or distance between microbiota sample pairs) of samples stored with and without glycerol was compared. When visualizing sample clustering by NMDS, no obvious clustering (eg, closeness of symbols and colors representing samples from donor and recipient at different time points) was evident (Figure 3, upper panel). The PERMANOVA was used to test multivariate community‐level differences between samples with and without glycerol, with individual included as strata, and sample type (FS = fecal sample before processing; FMT = prepared FMT slurry) and origin (donors or recipient) included as covariates. Sample origin was significantly correlated with differences in the microbiota (*P* = .03), but sample type (regardless of native FS or prepared FMT slurry) was not (*P* = .83). Glycerol addition was found to be significantly correlated with differences in microbiota composition (*P* ≤ .001). No interaction effects were observed between the covariates. Alpha diversity (within‐sample diversity or observed richness by number or evenness of taxa) also was measured by the inverse Simpson's index and Chao1 estimator (Figure 4A,B), but was not found to be significantly different between sample types.

The ANCOMBC was used to test whether individual OTUs and genera were significantly different between glycerol treated and untreated samples, including individual as a random effect, and sample type (FMT or FS) and sample origin as fixed effects. Two OTUs were identified as significantly more abundant in glycerol‐treated samples vs nontreated samples (Figure 4C): OTU 13 (*Fusobacteriaceae_unclassified* sp.) and OTU 98 (*Fusobacterium* sp.). Three genera were found to be more abundant in glycerol treated samples: *Butyricicoccus*, *Fusobacteriaceae_unclassified*, and *Helicobacter* (Figure 4D).

### 3.3 Changes of the fecal microbiota in FMT recipient dogs

**FIGURE 5:**
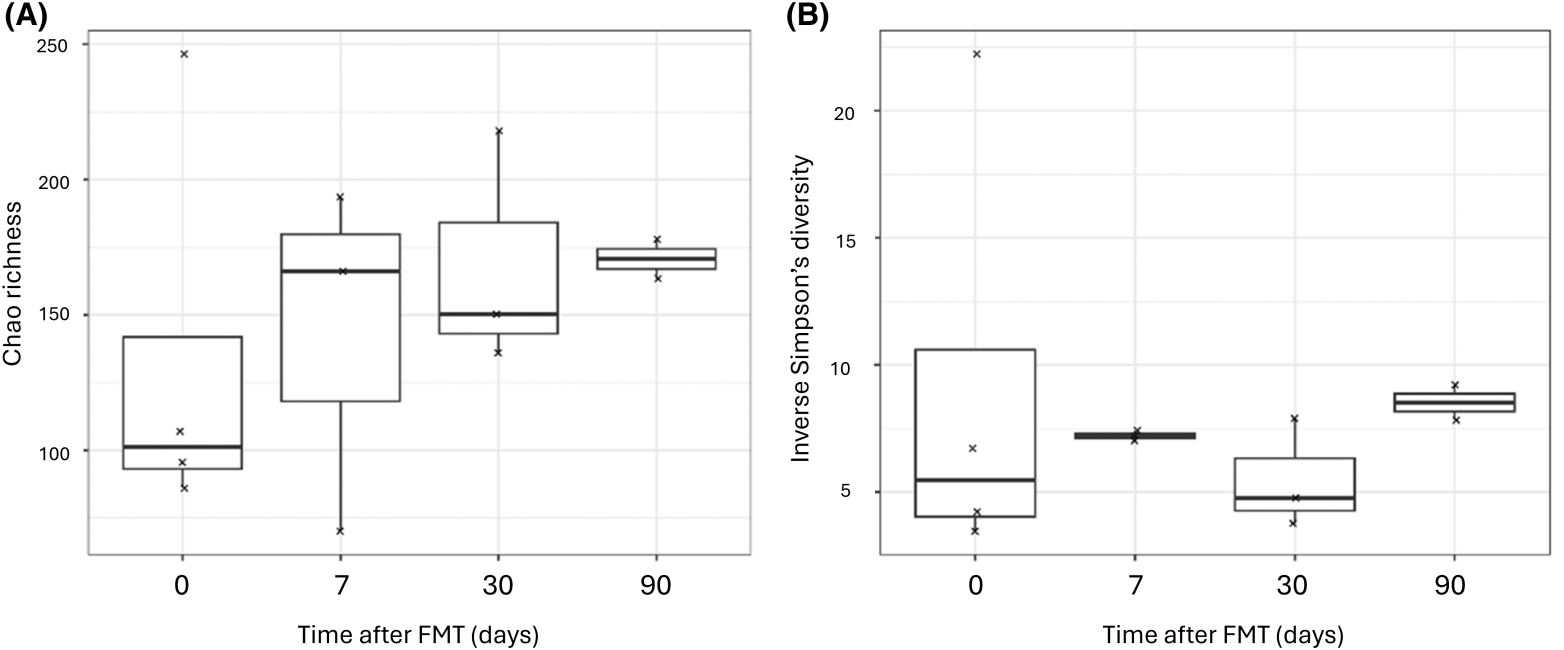
Boxplots showing richness (A: Chao1 Index) and diversity (B: Inverse Simpson's index) of fecal samples from dogs that had received 1 fecal transplant. FMT, fecal microbial transplant.

The PERMANOVA was used to test multivariate community‐level differences between samples from FMT recipients at different timepoints post FMT (Days 0, 7, 30, and 90) as available, with individual included as strata, and donor sample as a covariate. No significant differences were detected between any sample groups or covariates (*P* = .3), although, subjectively, recipient samples from before FMT (Day 0, Figure 3, lower panels, black data points) showed less similarity to donor samples (gray data points in Figure 3) compared with later time points. From Days 30 to 90 after FMT for donor 1 (Figure 3, lower left panel), donor and recipient samples clustered together, and were more distinct from the samples from before FMT. The effect was less evident for recipients of FMT from donor 2 (Figure 3, lower right panel). No consistent differences across samples were observed in alpha diversity for both donors combined (Chao *P* = .48, inverse Simpson *P* = .9; Figure 5).

**FIGURE 6:**
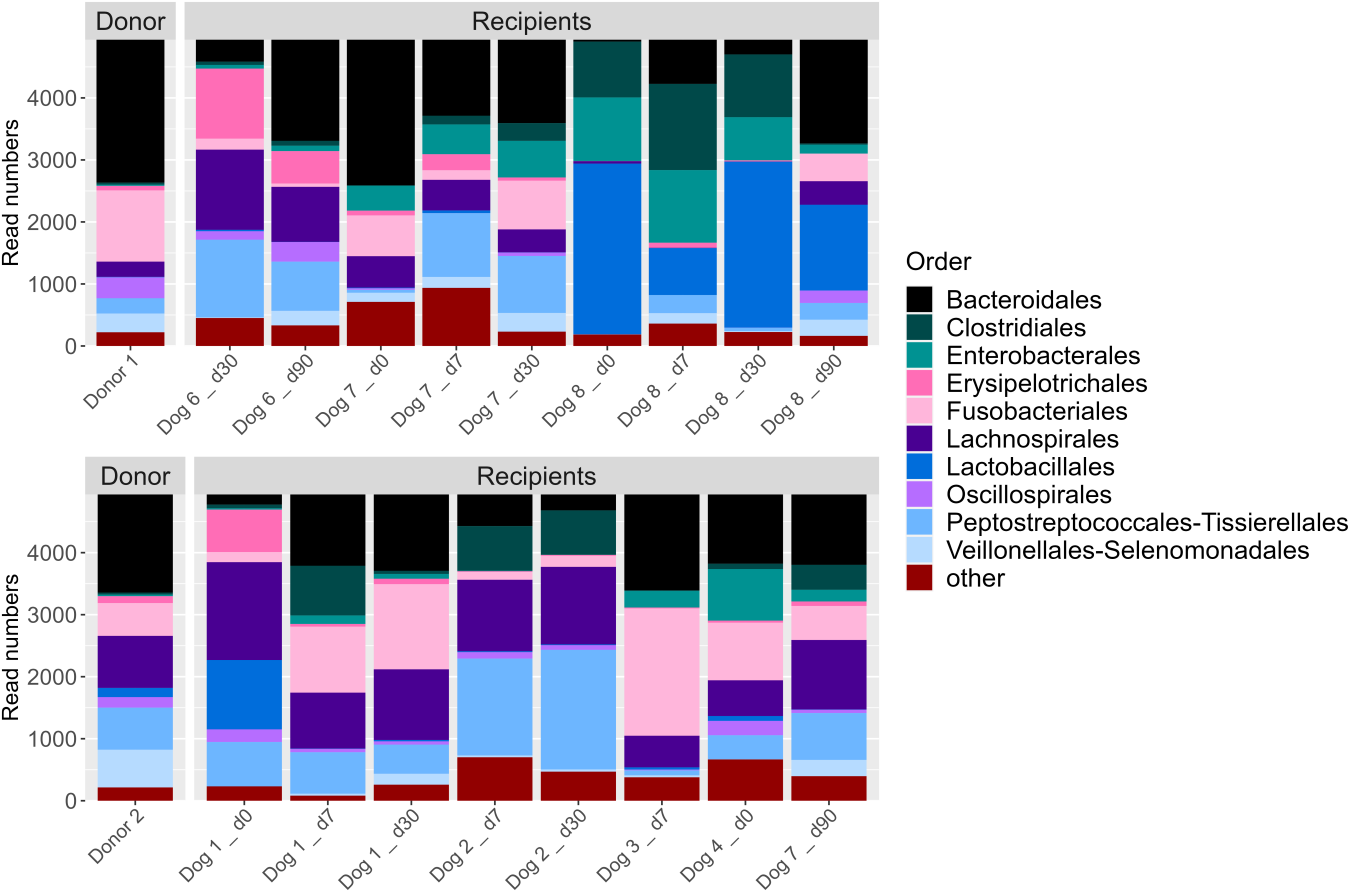
Barplot showing relative abundance of bacterial orders within fecal samples from FMT recipients at different time points after FMT and their matching donor.

**FIGURE 7:**
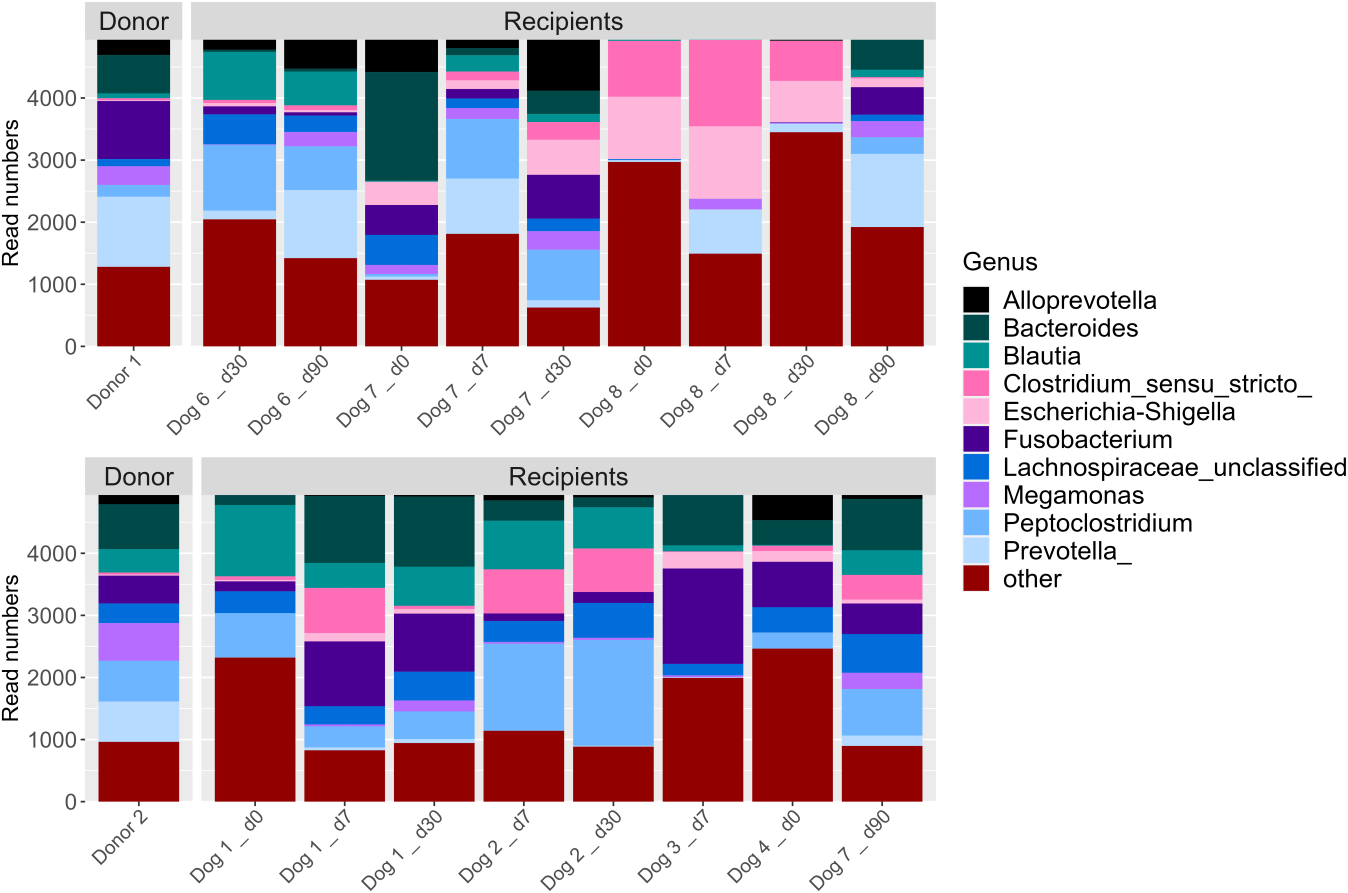
Barplot showing relative abundance of bacterial genera within fecal samples from FMT recipients at different time points after FMT and their matching donor.

Figures 6 and 7 show the relative abundance of bacterial orders and genera, matching donor samples to each recipient. Recipients did not show an approximation or similarity to their respective donors.

**FIGURE 8:**
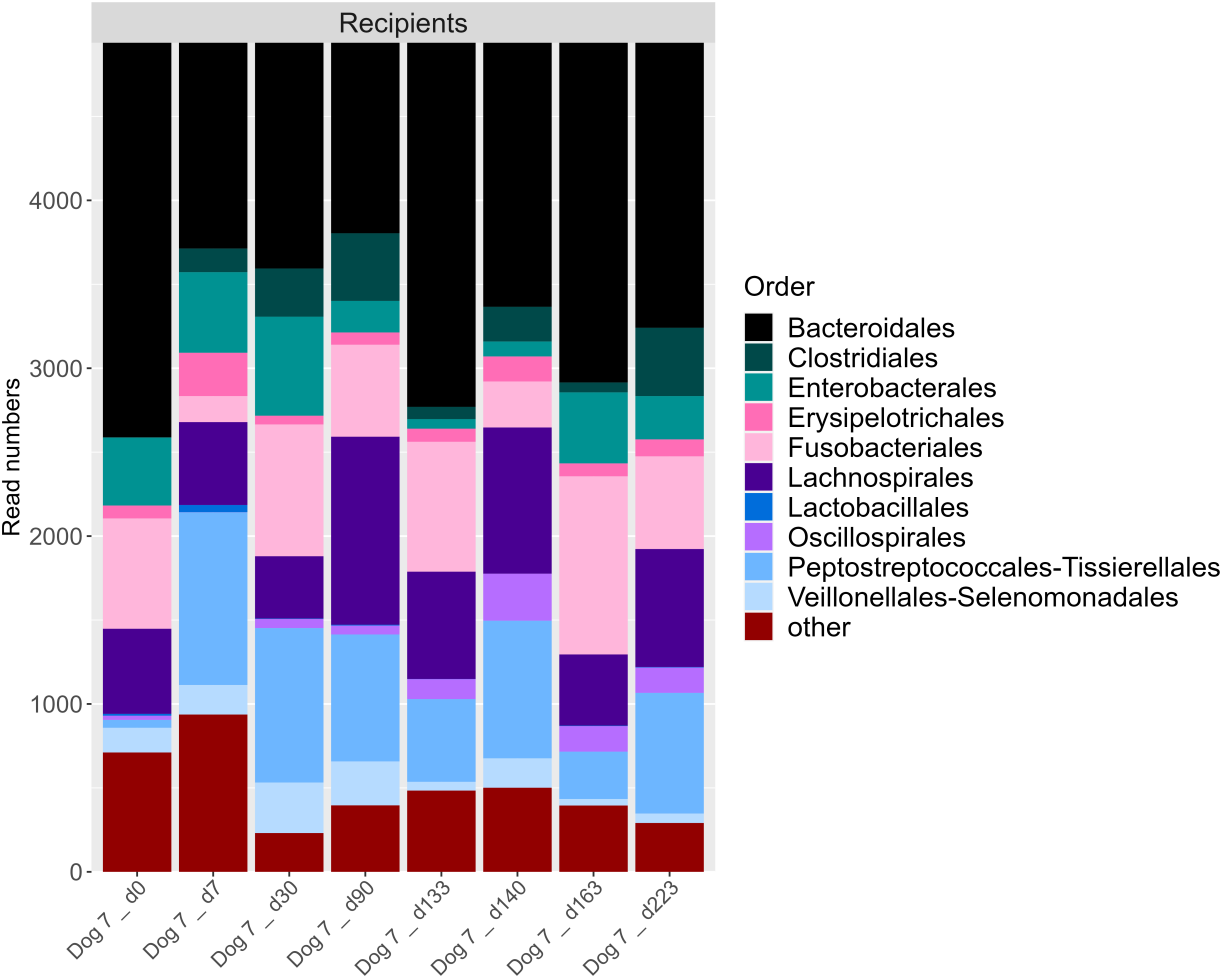
Barplot showing relative abundance of bacterial orders within fecal samples from 1 dog given 3 FMT treatments (from 2 different donors).

For the dog that received 3 sequential FMTs (Dog 7), we assessed microbiota composition after each FMT (first and third from donor 1, second from donor 2) and compared it to the respective donor at each donation. At both the order (Figure 8) and genus level the recipient microbiota did not resemble the donor microbiota composition.

## 4 DISCUSSION

We report both the assessment of clinical data and microbiota changes in dogs with CE that received a single FMT as an enema. Although in most dogs from this small cohort, a clinical benefit was evident within a short period of time (1 week), the duration of response was variable and of moderate duration (on average approximately 10 weeks). Only 1 of the dogs received repeated FMTs to control disease, in the other dogs, caretakers resorted to more traditional treatments, and a response or repeated FMTs could not be assessed. From a clinical perspective it hence remains unproven that FMT can be used as a sole long‐term treatment for CE, but repeated FMTs can be considered as part of a multimodal treatment approach to maintain or further improve clinical signs in individual dogs. The dog that received repeated FMTs had a variable response that could not be predicted by previous responses and might have been associated with the variation in FMT donors as well as other factors. We did not use the commercially available DI as part of the microbiota assessment of the recipients before and after FMT, as done in a recent study, and hence could not assess the level of individual eubiosis reached with a single FMT with this tool.^14^ The previous study demonstrated that the DI can be a potential predictor of clinical response, and results of this test are available within a reasonable period of time. However, for the 16S rRNA gene sequencing performed here, rapid availability of individual results is not possible, making it unsuitable as a clinical decision‐making tool to assess which dog might have benefited from >1 FMT. However, it has not been established that full engraftment of the donor microbiota or reestablishment of a normal microbiota composition as determined by the DI or other measures of microbiota composition, richness or function is necessary in dogs to achieve clinical responses. In fact, the data presented here suggests the opposite. In contrast to another study,^14^ clinical improvement can be observed despite a lack of microbiota changes, suggesting that clinical response could be driven by other factors such as microbiota metabolic products (so‐called postbiotics) or an interaction with the local mucosal immune system that does not require engraftment. In addition, we demonstrated that dogs with different severity of clinical signs based on CCECAI can react favorably to FMT, and thus FMT should not be reserved only for difficult to control cases or dogs with suspected NRE. In our study, a single FMT was planned to assess responses, and dog owners afterwards were given the choice to have FMT repeated or to revert to more traditional treatments for CE. As described above, most dog owners decided to not have FMT repeated (apart from the owners of Dog 7). Instead, previous acceptable or alternative treatments were instituted (eg, tylosine, which is no longer part of the standard treatment for CE).^33^ Consequently, the effect of repeated FMT, as done in some recent studies,^14^, ^34^ could not be assessed.

As stated, we could not demonstrate a consistent approximation of the recipient and donor microbiota profiles (neither in richness nor composition), which could be a consequence of many factors: donor selection was arbitrary and based on clinically assessable variables only, because our study was performed before publication of guidelines for FMT in companion animals.^35^ Specific microbiota characteristics were not included to select a donor (beyond excluding carriage of the most common parasitic and bacterial pathogens). Because there seems to be a potential but subjective difference between the 2 donors used, it is possible that improvement in the selection criteria for donors could result in better clinical responses of the recipients. Little information is available on day‐to‐day variability of FMT donor microbiota, and this factor should be explored further in the future. Finally, the number of recipient dogs was relatively small, and it is possible that patterns would emerge with a larger number of dogs included.

The relatively short‐term and overall low rate of complete clinical response to FMT in dogs with CE is similar to what is observed in people with CD (approximately 30% clinical response),^36^ which is not as high as with infectious disease (eg, *Clostridium difficile* infection in people^37^ and parvovirosis in dogs^12^). It seems feasible to expect similar results with chronic multifactorial conditions such as CE, because only 1 of the potential aspects driving inflammation (eg, the microbiota) is modulated, whereas others (genetic risk factors, environmental influences, mucosal immune response) remain difficult to target and likely unchanged by FMT, especially if more permanent engraftment of healthy donor microbiota cannot be achieved. A recent study in a larger cohort of dogs receiving multiple FMTs showed a more consistent shift in microbiota composition (albeit only using the DI),^14^ and thus it would be pertinent in future studies to thoroughly define treatment protocols and endpoint assessments to develop a more standardized FMT protocol as adjunctive treatment for CE in dogs.^38^

The criteria by which to select CE recipients that have the highest likelihood of favorable responses to FMT also is unknown in dogs. Follow‐up studies using recently available guidelines for donor selection and FMT administration protocols^35^ likely will determine if a more intensive FMT protocol and more careful selection and monitoring of recipients with more readily available tools might be needed to improve clinical, microbiota and other as of yet undefined outcome measures in dogs.

Because the FMTs used for our study were given immediately after preparation, considerations for FMT slurry storage were not of immediate relevance regarding FMT success for these specific recipients. However, because the addition of glycerol frequently is promoted when storing FMT for both people and dogs,^39^, ^40^ this procedure is important to veterinary practitioners who wish to use FMT regularly with the potential option for storage. Although a recent study on FMT in dogs with CE showed clinical efficacy without the addition of glycerol,^14^ there are microbiological advantages to adding a cryopreservative.^41^ For FMT preparations used in dogs, we showed that storage with 10% glycerol preserved a small number of bacterial species at higher levels than without the glycerol. Although 1 genus (*Butyricicoccus* sp.) is a butyrate‐producing bacterium,^42^ and hence potentially beneficial, its individual importance and clinical relevance for successful FMT treatment in dogs is unknown. *Butyricicoccus pullicaecorum* recently has been found to be depleted in people with inflammatory bowel disease, and correlated with disease activity in CD.^43^ Moreover, PO administration of *B. pullicaecorum* resulted in a significant protective effect from drug‐induced colitis in a rat model.^43^

In conclusion, a single FMT can be considered an appropriate part of the multimodal treatment approach in dogs with CE, and achieves short‐term clinical improvement when performed once. Consistent microbiota changes more closely resembling healthy donors, however, were not observed in our study. Although additional prospective studies using standardized processes for FMT preparation, administration, and monitoring as well as recipient selection are needed, our results represent a promising start for microbiota‐targeted treatments more routinely as part of CE treatment in dogs. Storage of FMT preparations in glycerol should be considered to preserve microbiota composition, but how doing so would affect clinical efficacy is unclear. A recent study in a larger cohort of dogs demonstrated that fecal donations stored without glycerol have sufficient clinical effect.^14^ Future studies addressing optimal storage requirements for donations and FMT preparations that allow updating of available guidelines^35^ still are required.

## CONFLICT OF INTEREST DECLARATION

Authors declare no conflict of interest.

## OFF‐LABEL ANTIMICROBIAL DECLARATION

Authors declare no off‐label use of antimicrobials.

## INSTITUTIONAL ANIMAL CARE AND USE COMMITTEE (IACUC) OR OTHER APPROVAL DECLARATION

Authors declare no IACUC or other approval was needed.

## HUMAN ETHICS APPROVAL DECLARATION

Authors declare human ethics approval was not needed for this study.

## Supporting information


**Table S1.** Signalment and clinical data of the 7 dogs with CE receiving FMT. ARE, antibiotic‐responsive enteropathy; CYC, cyclosporine; DSF, de Simone formulation probiotics; EO, eosinophilic; F, female; FB, foreign body; GID, gastrointestinal diet; INA, inappetence; IRE, immunosuppressive‐responsive enteropathy; HD, hydrolyzed diet; LBD, large bowel diarrhea; LPC, lymphoplasmacytic; LTH, lethargy; MET, metronidazole; MN, male neutered; NPD, novel protein diet; NRE, nonresponsive chronic enteropathy; Pred, prednisolone; SBD, small bowel diarrhea; TYL, tylosin; V, vomiting; WL, weight loss; WNL, within normal limits.
