# Supplementary material for: Effect of a single rectal fecal microbiota transplantation on clinical severity and fecal microbial communities in dogs with chronic inflammatory enteropathy
Source: J Vet Intern Med. 2025 Jan 8;39(1):e17264. doi: 10.1111/jvim.17264 (PMC11710856; doi:10.1111/jvim.17264)
Supplement: Supplementary file 1 — Table S1. Signalment and clinical data of the 7 dogs with CE receiving FMT. ARE, antibiotic‐responsive enteropathy; CYC, cyclosporine; DSF, de Simone formulation probiotics; EO, eosinophilic; F, female; FB, foreign body; GID, gastrointestinal diet; INA, inappetence; IRE, immunosuppressive‐responsive enteropathy; HD, hydrolyzed diet; LBD, large bowel diarrhea; LPC, lymphoplasmacytic; LTH, lethargy; MET, metronidazole; MN, male neutered; NPD, novel protein diet; NRE, nonresponsive chronic enteropathy; Pred, prednisolone; SBD, small bowel diarrhea; TYL, tylosin; V, vomiting; WL, weight loss; WNL, within normal limits. [file JVIM-39-e17264-s001.docx]

**Supplementary Table S1.** Signalment and clinical data of the 7 dogs with CE receiving FMT. CYC = cyclosporine, DSF = de Simone formulation probiotics, EO = eosinophilic, FB = foreign body, GID = gastrointestinal diet, INA = inappetence, IRE = immunosuppressive-responsive enteropathy, HD = hydrolyzed diet, LBD = large bowel diarrhea, LPC = lymphoplasmacytic, LTH = lethargy, MET = metronidazole, NPD = novel protein diet, NRE = non-responsive enteropathy, Pred = prednisolone, SBD = small bowel diarrhea, V = vomiting, WL = weight loss, WNL = within normal limits

| **Recipient number** | **Breed** | **Age (months)** | **Gender** | **Main presenting complaint** | **Previous (failed) treatments** | **Histopathology findings** | **CE subtype** |
| --- | --- | --- | --- | --- | --- | --- | --- |
| 1 | Staffordshire Terrier cross | 106 | MN | LBD > SBD, INA & LTH for 5 years, perianal dermatitis | GID, 2 x HD, EF, MET, TYL, Pred, CYC | Mild, diffuse LPC colitis | NRE (partial response to Pred) |
| 2 | Pekingese | 77 | MN | LBD> SBD, intermittent V, pruritic skin disease, otitis externa | GID, MET, TYL, NPD, Pred | Minimal LPC gastritis, moderate diffuse LPC and EO duodenitis, ileitis and colitis | NRE, (partially FRE, “bouts” of ARE, intermittent partial response to Pred) |
| 3 | Irish Setter | 43 | F | INA, V, intermittent SBD | GID, 2 x NPD, TYL | Mild LPC gastritis, moderate LPC duodenitis, mild LPC ileitis, minimal LPC colitis | ARE (best response to Tylosin) |
| 4 | German Shepherd Dog | 88 | MN | SBD, WL | NPD, MET, TYL, Pred | WNL (stomach, duodenum, colon) | IRE |
| 5 | Whippet | 42 | MN | V, SBD > LBD, pruritic skin disease | HD, NPD, HD, DSF, TYL, MET, Pred | Stomach WNL, moderate diffuse EO duodenitis and ileitis, mild diffuse LPC colitis with fibrosis and edema | IRE |
| 6 | Labrador Retriever | 5 | F | Acute D with FB turned into chronic LBD > SBD with INA and LTH, abdo pain, possible melena, WL | GID, NPD, TYL | Mild LPC & EO gastritis, Mild to moderate LPC & EO duodenitis and ileitis, mild LC and EO colitis | ARE |
| 7 | Leonberger | 90 | MN | V, pica, SBD | NPD, MET | Minimal multifocal EO gastritis, mild to moderate diffuse LPC & EO duodenitis and ileitis, colon WNL | ARE |
